# Integrated efforts to promote mental health care during the SARS-CoV-2 pandemic: Reflecting on the experience of a university helpline

**DOI:** 10.1192/j.eurpsy.2021.749

**Published:** 2021-08-13

**Authors:** A. Torres, I.M. Santos, C. Rosa, S. Monteiro, F. Rodrigues, A. Figueiredo, T. Santos, O. Ribeiro, A. Queirós, A. Pereira, C. Silva

**Affiliations:** 1 Department Of Education And Psychology Of University Of Aveiro, CINTESIS - UA – Center for Health Technology and Services Research, Portugal (R&D Unit ref. UIDB/4255/2020), Aveiro, Portugal; 2 Department Of Education And Psychology Of University Of Aveiro, William James Center for Research, Aveiro, Portugal; 3 Department Of Education And Psychology, University of Aveiro, Aveiro, Portugal; 4 Department Of Education And Psychology Of University Of Aveiro, Research Centre in Didactics and Technology in the Education of Trainers (CIDTFF), Aveiro, Portugal; 5 Department Of Mental Health, CHEDV - Centro Hospitalar do Baixo Vouga, Aveiro, Portugal; 6 Rectorate, University of Aveiro, Aveiro, Portugal

**Keywords:** university helpline, psychological intervention in crisis, SARS-CoV-2, integrated mental health care

## Abstract

**Introduction:**

The SARS-CoV-2 pandemic is affecting numerous dimensions of our society since the beginning of the outbreak. A significant increase in emotional distress was expected in the general population, particularly among the high-risk groups such as the oldest, chronic patients, healthcare professionals, and psychopathology vulnerable people. There was an urgent need to adapt and create solutions to promote mental health. Given the recommendations to minimize face-to-face interactions, several helplines were widely developed.

**Objectives:**

In this work, we aim to reflect on the experience of a university helpline, that integrated efforts with the regional mental health care services.

**Methods:**

A University helpline was created to give support to the regional community outside academia. The team was created on an online teamwork platform, to communicate through the chat, carry videoconference meetings, and store useful files. A Manchester screening decision tree was adopted, to define a set of guidelines to provide support to the callers, based mainly on the guidelines defined by the Order of Portuguese Psychologists. Liaison with the mental health care services, including other specific helplines, was established.

**Results:**

Notwithstanding all the efforts, the number of received calls was scarce, similarly to helplines created by other national universities and by other entities.

**Conclusions:**

A new approach to psychological intervention in crisis is needed, maintaining integrated efforts, and taking advantage of the opportunity to foster personalized mental health care in the digital era. It is important to continuously assess the value of integrated efforts in patient care and to the healthcare system.

